# The practice and evaluation of antifungal stewardship programs at a tertiary first-class hospital in China

**DOI:** 10.1186/s12879-024-09405-x

**Published:** 2024-05-21

**Authors:** Huiyuan Zhang, Yinglin Wang, Ruigang Diao, Xuechen Huo, Quan Zhao

**Affiliations:** 1 Department of Pharmacy, Yantai Yuhuangding Hospital, Yantai, Shandong Province China; 2 Department of Hepatobiliary Surgery, Yantai Yuhuangding Hospital, Yantai, Shandong Province China

**Keywords:** Antifungal stewardship, Plan-do-check-act, Antifungal drug consumption, Proportion of rational prescriptions, Drug resistance of fungi

## Abstract

**Background:**

The sharp increase in fungal infections, insufficient diagnostic and treatment capabilities for fungal infections, poor prognosis of patients with fungal infections as well as the increasing drug resistance of fungi are serious clinical problems. It is necessary to explore the implementation and evaluation methods of antifungal stewardship (AFS) to promote the standardized use of antifungal drugs.

**Methods:**

The AFS programme was implemented at a tertiary first-class hospital in China using a plan-do-check-act (PDCA) quality management tool. A baseline investigation was carried out to determine the utilization of antifungal drugs in pilot hospitals, analyse the existing problems and causes, and propose corresponding solutions. The AFS programme was proposed and implemented beginning in 2021, and included various aspects, such as team building, establishment of regulations, information construction, prescription review and professional training. The management effectiveness was recorded from multiple perspectives, such as the consumption of antifungal drugs, the microbial inspection rate of clinical specimens, and the proportion of rational prescriptions. The PDCA management concept was used for continuous improvement to achieve closed-loop management.

**Results:**

In the first year after the implementation of the AFS programme, the consumption cost, use intensity and utilization rate of antifungal drugs decreased significantly (*P* < 0.01). The proportion of rational antifungal drug prescriptions markedly increased, with the proportion of prescriptions with indications increasing from 86.4% in 2019 to 97.0% in 2022, and the proportion of prescriptions with appropriate usage and dosage increased from 51.9 to 87.1%. In addition, after the implementation of the AFS programme, physicians’ awareness of the need to complete microbial examinations improved, and the number of fungal cultures and serological examinations increased substantially. Statistics from drug susceptibility tests revealed a decrease in the resistance rate of *Candida* to fluconazole.

**Conclusion:**

This study indicated that the combination of AFS and the PDCA cycle could effectively reduce antifungal consumption and promote the rational use of antifungal drugs, providing a reference for other health care systems to reduce the overuse of antifungal drugs and delay the progression of fungal resistance.

**Supplementary Information:**

The online version contains supplementary material available at 10.1186/s12879-024-09405-x.

## Background

The resistance of microorganisms has become a severe public health crisis, resulting in increased patient mortality and medical costs [1]. Governments worldwide have continued to launch major measures to promote the rational use of anti-infective drugs and curb bacterial resistance. Antimicrobial stewardship (AMS) is an internationally recognized strategy proposed in 1996 to establish a scientific management system around “how to choose”, “how to manage” and “how to use” antibiotics in hospitals to address the global crisis of bacterial resistance [2]. In the past 30 years, the global practice of AMS has shown that measures such as promoting guidelines, enhancing the microbiological inspection rate, and reviewing prescriptions could substantially improve the quality of antimicrobial application and reduce the trend of bacterial resistance [3, 4].

In the past few years, with advances in medicine, the increasing number of immunocompromised patients, the widespread use of broad-spectrum antibacterial drugs and so on, the incidence rate of invasive fungal diseases (IFDs) has substantially increased [5]. IFDs are associated with high patient mortality, and the expensive cost of antifungal drugs has a large economic impact worldwide. In addition, the varying risk of IFDs among different patients, as well as subtle differences in the pharmacokinetics and pharmacodynamics of a variety of antifungal drugs, further increase the complexity of treating fungal infections [6]. For these reasons, antifungal drugs must be used appropriately based on the full understanding of their indications, activities, safeties, and pharmacokinetics. Antifungal stewardship (AFS) emerged to optimize the use of antifungal drugs by integrating the knowledge and experiences of multidisciplinary teams, which aimed to ensure the suitable selection of antifungal drugs with optimized dosages, application methods and treatment courses [7].

AFS shares similar goals with AMS, such as promoting the scientific and rational use of antimicrobial drugs, appropriately simplifying the prescription of antifungal drugs, and preventing the spread of multidrug-resistant microorganisms [8]. However, there are several unique difficulties in implementing AFS due to the differences between bacterial infection and fungal infection [9–11]. (I) Patients infected with bacteria could be either inpatients or outpatients, while fungal infections mostly occur in immunosuppressed hospitalized patients. (II) Most clinicians are experienced in the treatment of bacterial infections, but there is often a knowledge gap among clinicians regarding the treatment of IFDs. (III) There is little diversity and poor accessibility of diagnostic methods for fungal infection, and these methods have not been developed in many primary hospitals. (IV) Compared with antibacterial drugs, antifungal drugs have few varieties, and most of them have the disadvantages of high price, remarkable adverse drug reactions, multiple drug interactions and long treatment courses, which could cause heavy economic burdens for patients.

Therefore, there is an urgent need to establish a management system for antifungal drugs in pilot hospitals, explore the replicable scientific practice methods for AFS with formalized inspection methods and evaluate the effects of AFS after its implementation. Some health care organizations have recognized the necessity of antifungal management programs, and there have been certain reports showing the positive role of AFS [12, 13]. However, the specific intervention measures and their effectiveness in various aspects were still unclear and need to be further systematized and standardized. The plan-do-check-act (PDCA) cycle proposed by Dr Edwards Demingin in the 1950s has been widely used for the continuous improvement of medical quality management. To our knowledge, few studies have reported the use of the PDCA cycle for the scientific management of antifungal drugs. Here, we proposed and implemented AFS combined with the PDCA cycle in a pilot tertiary first-class hospital in China beginning in 2021 to explore practical measures suitable for the standardized use of antifungal drugs in hospitals and to continuously improve the use of antifungal drugs.

## Methods

### Plan

#### Study setting and patient population

This study was a prospective interventional study conducted at a 4000-bed tertiary first-class teaching hospital in China and specifically tailored to evaluate the direct impacts of AFS measures on the rationality of antifungal drug use. This hospital is a regional medical centre covering solid organ transplants, heart surgery, stem cell transplants and intensive medical care. The antifungal susceptibility tests of *Candida* are performed based on the microdilution method using the commercial ATB FUNGUS-3 kit according to the latest Clinical and Laboratory Standards Institute (CLSI) M27, M59, and M60 standard methods, while the susceptibility testing has not carried out for filamentous fungi [14–18].

The main outcome measure for this study was the rationality of antifungal drug use, evaluated by the consumption of antifungal drugs (cost, use intensity and utilization rate), the rational rate of antifungal drug prescriptions (proportion with indications, proportion with appropriate usage and dosage), and the microbiological examination before the application of antifungal drugs. Other outcome measures included the composition of antifungal drug consumption, the composition of fungi detected, as well as the resistance rate of *Candida*. The data were collected over a 4-year period, starting in January 2019 and ending in December 2022. AFS was implemented beginning in January 2021. The baseline data in January 2019-December 2020 were extracted as comparison groups to conduct the before-after control analysis with the data after the AFS intervention (January 2021-December 2022). The study population included all inpatients admitted to this pilot hospital during the study period, covering both adults and paediatric patients. This study was approved by the Ethics Committee of Yantai Yuhuangding Hospital.

#### Baseline data collection and problem analysis

Before the implementation of AFS, the baseline data on the use of antifungal drugs were investigated from the perspectives of hospitals and departments. Data on the use of antifungal drugs were extracted directly from the large database of the hospital information system (HIS) and the electronic medical records of patients (2019–2022), recorded in terms of the utilization rate (%), the consumption cost (Chinese yuan), and the use intensity (defined daily doses/100 patient-days) [19]. The personal information of the patients was hidden. The number of microbial tests and drug resistance data were kindly provided by the microbiology laboratory. The reasonableness of the use of antifungal drugs was evaluated based on the comments of a large number of prescriptions.

#### Cause analysis and solutions

The use of various antifungal drugs before AFS was analyzed in pilot hospital and main reasons for the unreasonable use of antifungal drugs were summarized. In view of these, the establishment of an AFS management team was proposed for identifying the responsibilities and obligations of each member. Professional knowledge training on antifungal drugs was strengthened for medical staff. The preprocessing of antifungal drug prescriptions was strengthened based on the information system. In addition, the management system for antifungal drugs was made sound, with clear reward and punishment measures.

### Do

#### Establishment of the AFS management team

The AFS management team was formed to be responsible for the construction and implementation of the antifungal drug management system, with the hospital director as the primary responsible person. The team was composed of members of the medical affairs department, the department of pharmacy, the infectious disease department, the microbiology laboratory, the nursing department, the infection control department, the quality control department, the network information management office, medical imaging department and key clinical departments (the department of haematology, the department of respiratory medicine, the department of organ transplantation, department of critical medicine and the department of pediatrics). The medical affairs department and the pharmacy department were jointly responsible for daily management. The main responsibility of the management team covered the top-level design and promotion of the entire project, including the baseline investigation, problem summarization and analysis, development of management measures, formulation of assessment indicators, and the final acceptance of the project, which followed the PDCA principles. Multiple departments involved in building the AFS management team worked together to improve the standardized use of antifungal drugs.

#### Building an AFS professional technical team

The core members of this project mainly included infectious disease physicians, clinical pharmacists specializing in anti-infective agents and microbiologists who were experienced in the treatment of IFDs. These individuals were responsible for the technical guidance and consultation on the clinical use of antifungal drugs in clinical departments and provided professional training related to the clinical use of antifungal drugs for medical staff. For difficult and complex cases of fungal infection such as the decisions about antifungal prevention on immunosuppressed patients with risk for drug interactions, multidisciplinary consultations of expert team were organized regularly to determine the most appropriate treatment strategy.

#### Rules and regulations

In accordance with the spirit and requirements of national policies and regulations, the AFS management team formulated and updated the management regulations on the use of antifungal drugs in the medical institution in a timely manner. For example, the pilot hospital developed a hierarchical management system for antifungal drugs to restrict the prescription of antifungal drugs for physicians. In addition, a comprehensive evaluation system has been established based on the participation rates in AFS training, examination scores, formulation of diagnostic and treatment standards, rational rates of prescriptions, microbial examination rates, and the improvement of AFS management indicators for each department. This system could not only serve as an important basis for the performance assessment and promotion of professional titles of responsible physicians; but also was linked to the assessment and punishment of relevant departments.

#### Formulation of guidelines for antifungal treatment at the medical institution

Early diagnosis of IFDs and identification of indications for antifungal drugs are important factors affecting patient prognosis. The rationality of the antifungal drugs evaluated in this study was based mainly on the guidelines formulated by the Infectious Diseases Society of America (IDSA) [20, 21] and the European Society of Clinical Microbiology and Infectious Diseases (ESCMID) [22, 23], as well as some treatment guidelines in China [24–26]. Departments with high consumption of antifungal drugs were required to develop norms for the diagnosis and treatment of fungal infectious diseases applicable to their own departments.

#### Education and training

Experts outside and within the hospital were regularly invited to carry out professional training for clinical departments to strengthen awareness of the rational use of antifungal drugs, standardize empirical drug use, and reduce the use of antifungal drugs without indications. The superintendent and representative physician in each department were required to attend the above trainings.

#### Information construction

Information technology was fully utilized for the precontrol of the prescription of antifungal drugs. By directly writing rules and self-defining knowledge bases, we could achieve the pre-examination of indications and the interception of unreasonable drug use, and prompt microbiological inspection.

#### Implementation of standardized diagnosis and screening for fungal infections

To improve the screening rate of fungal infections and avoid missed detections by clinicians who were unfamiliar with the relevant testing items, the microbiology laboratory innovatively carried out modular testing projects. Serological screening of *Candida* infection included the use of a 1,3-β-D-glucan (G) test, *Candida* antigen detection, and *Candida* IgG antibody detection. Serological screening of *Aspergillus* infection included the G test, galactomannan (GM) test and *Aspergillus* IgG antibody detection. These diagnostic methods were continuously optimized based on new technologies and research achievements.

#### Prescription review

Clinical pharmacists specializing in anti-infective agents were responsible for reviewing the rationality of antifungal drug prescriptions. According to the guidelines, IFDs were stratified and diagnosed as undetermined, possible, probable, or proven fungal infections based on clinical characteristics, radiological results, and microbiological findings. Corresponding antifungal treatments could be classified as prophylaxis therapy, empirical therapy, preemptive therapy or targeted therapy [26]. A rationality check form for the clinical application of antifungal drugs for patients was designed to collect relevant data and evaluate the rationality of antifungal prescriptions for specific cases. The review results were fed back to the clinical departments in the form of notifications. Relevant problems were analysed in detail at the hospital management meeting, and targeted training was conducted in the involved departments. In addition, the problems found in prescription reviews would be fed back to the information management system to constantly enrich the knowledge base and add new rules to avoid the recurrence of similar problems.

#### Periodic evaluation and optimization of the catalogues for antifungal drugs

According to the results of comprehensive clinical evaluation of the drugs, the drug purchasing department dynamically managed the variety and quantity of antifungal drugs. The list of antifungal drugs was regularly evaluated, adjusted and optimized. Clinically important agents with abundant evidence were introduced, while antifungal drugs with less consumption and uncertain clinical effects were classified as temporary drugs, ensuring the rational allocation of the antifungal drug catalogue.

#### Establishment of diagnosis and treatment centres for special diseases

For departments with a high incidence of fungal infections, regular multidisciplinary rounds were carried out. Clinical pharmacists, microbiologists, and infectious disease doctors worked together in clinical departments to establish multidisciplinary diagnosis and treatment centres for special diseases.

#### Monitoring and early warning

The pharmaceutical department was responsible for monitoring the use of antifungal drugs, focusing on the utilization rate, use intensity, and use trend of various antifungal drugs. Relevant information would be submitted to the Center for Antibacterial Surveillance of China as required. Moreover, the microbiology laboratory was responsible for monitoring the microbial detection, fungal epidemiology and drug resistance of the fungi. These results would be regularly submitted to the Fungal Diseases Surveillance System of China as required. Abnormal consumption of antifungal drugs and severe fungal resistance would be regularly monitored and reported, and targeted control measures would be formulated.

#### Discipline construction and personnel training

The construction of disciplines related to the clinical use of antifungal drugs was strengthened, covering the improvement and optimization of disciplinary structure, the development of new technologies and projects, and the introduction of equipment and talent. The role of relevant professionals in the clinical application management of antifungal drugs was fully explored.

### Check

The management team regularly conducted statistical analysis and made comments on the use of antifungal drugs in the entire hospital and in various departments to evaluate the effectiveness of AFS. The clinical application management indicators for AFS were developed and a periodic evaluation table of AFS management effectiveness for clinical departments was employed to score the rationality of antifungal drugs.

### Act

Clinical departments were asked to establish a “self-examination - verification - feedback - rectification” mechanism and submit the rectification report on time. The AFS working groups regularly organized antifungal drug management meetings to report the test results. The achievements and problems experienced during the previous stage were summarised, during which the problems were evaluated and rectified. The unsolved problems were transferred to the next PDCA cycle for solution to continuously optimize the management measures of antifungal drugs.

### Statistical analysis

Mean values and standard deviations (SD) were used for continuous variables with a normal distribution and median and interquartile range (IQR) was used if the data had a non-normal distribution. Categorical variables were expressed as percentages. SPSS20 was used for the statistical analysis. T-test was used to compare the consumption of antifungal drugs (cost, use intensity and utilization rate) before and after the implementation of AFS. The rational rates of antifungal drug prescriptions (proportion with different indications, proportion with appropriate usage and dosage), and the microbiological examination rates before and after the implementation of AFS were analysed by the chi-square (x^2^) test. All statistical tests were two-tailed. P value < 0.05 was considered to indicate statistical significance.

## Results

### Baseline data analysis

**Fig. 1:**
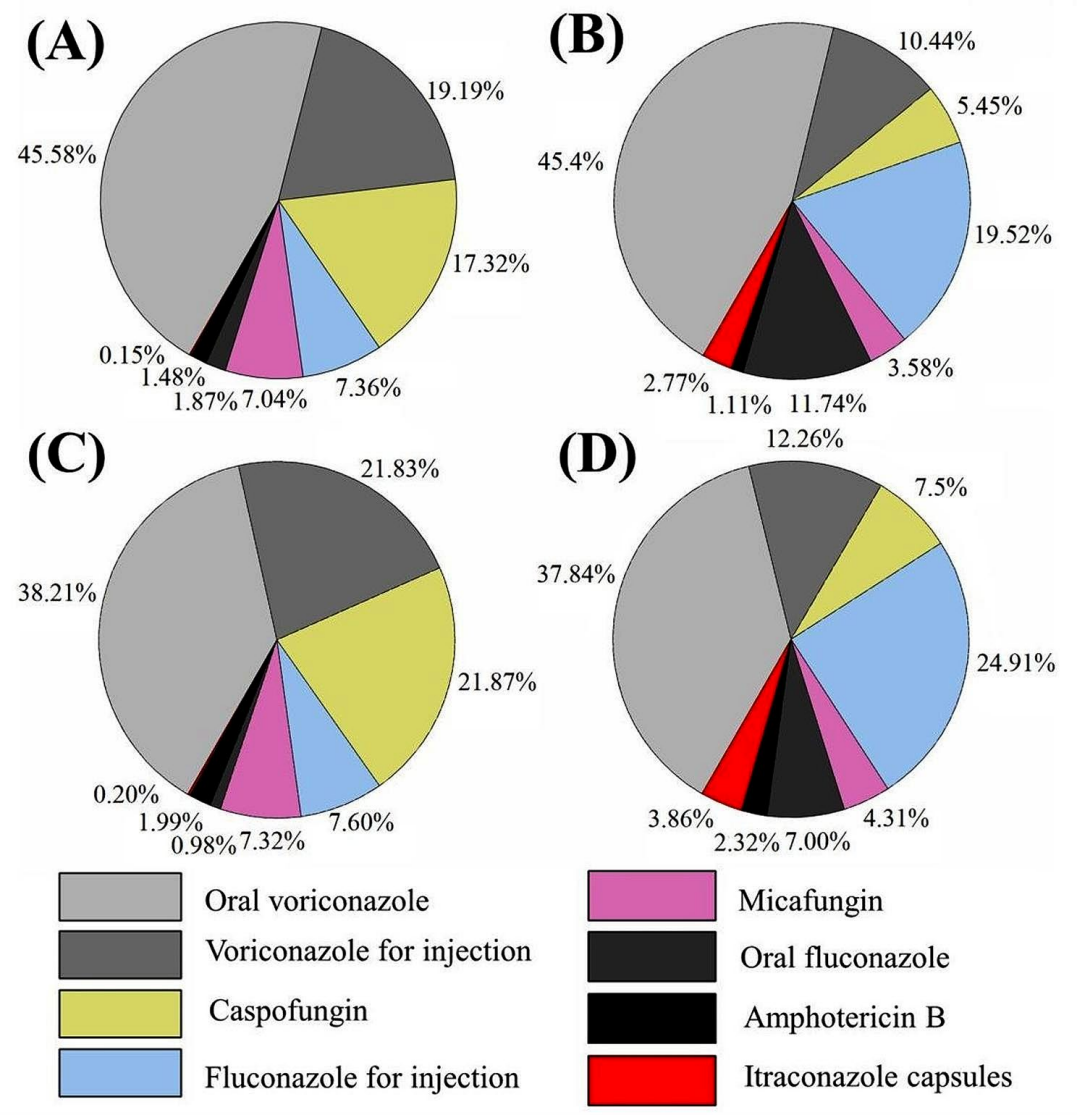
The proportion of consumption for different antifungal agents used in 2019 calculated in (**A**) cost and (**B**) the antifungal drug use intensity. The proportion of consumption for different antifungal agents used in 2020 calculated in (**C**) cost and (**D**) the antifungal drug use intensity

Before the intervention of AFS, the cost of antifungal drugs exceeded 10% of the total cost of antibacterial drugs in our hospital, with an average of 11.88 million Chinese yuan per year. As shown in Fig. 1, during the baseline survey period, the most commonly used antifungal drugs were oral voriconazole calculated by cost and antifungal drug use intensity. And voriconazole of different dosage forms accounted for more than 50% of the consumption of all antifungal drugs.

**Fig. 2:**
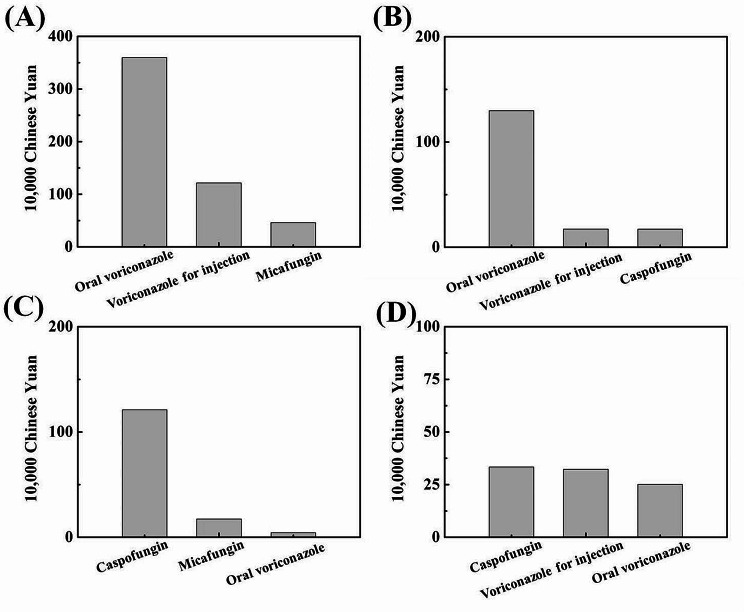
The top three antifungal drugs consumed in (**A**) the department of haematology, (**B**) the respiratory medicine department, (**C**) the department of organ transplantation and (**D**) the department of critical medicine ranked by cost in 2019

Figure S1 shows that the key departments with the greatest antifungal drug use were the department of haematology, the department of respiratory medicine, the department of organ transplantation and department of critical medicine. As shown in Fig. 2, Fig. S2 and Table S2, the consumption of different antifungal drugs, especially voriconazole was higher in the department of haematology compared with other departments before the AFS intervention, while the positive number of *Aspergillus* cultures, fluorescence staining microscopy and related serological tests was relatively low. In the respiratory medicine department, the most widely used antifungal drug was still voriconazole. The number of GM test-positive in BALF (bronchoalveolar lavage fluid) samples and the number of *Aspergillus* culture-positive samples was the highest in the counted key departments, and the number of GM test-positive in BALF samples was nearly 4 times greater than the number of *Aspergillus* culture-positive samples. In the department of organ transplantation, the total consumption cost of antifungal drugs was comparable to that in the department of respiratory medicine. The number of positive results from microbiological examinations was much lower than that in the department of respiratory medicine.

### Economic impact and potential cost savings

**Fig. 3:**
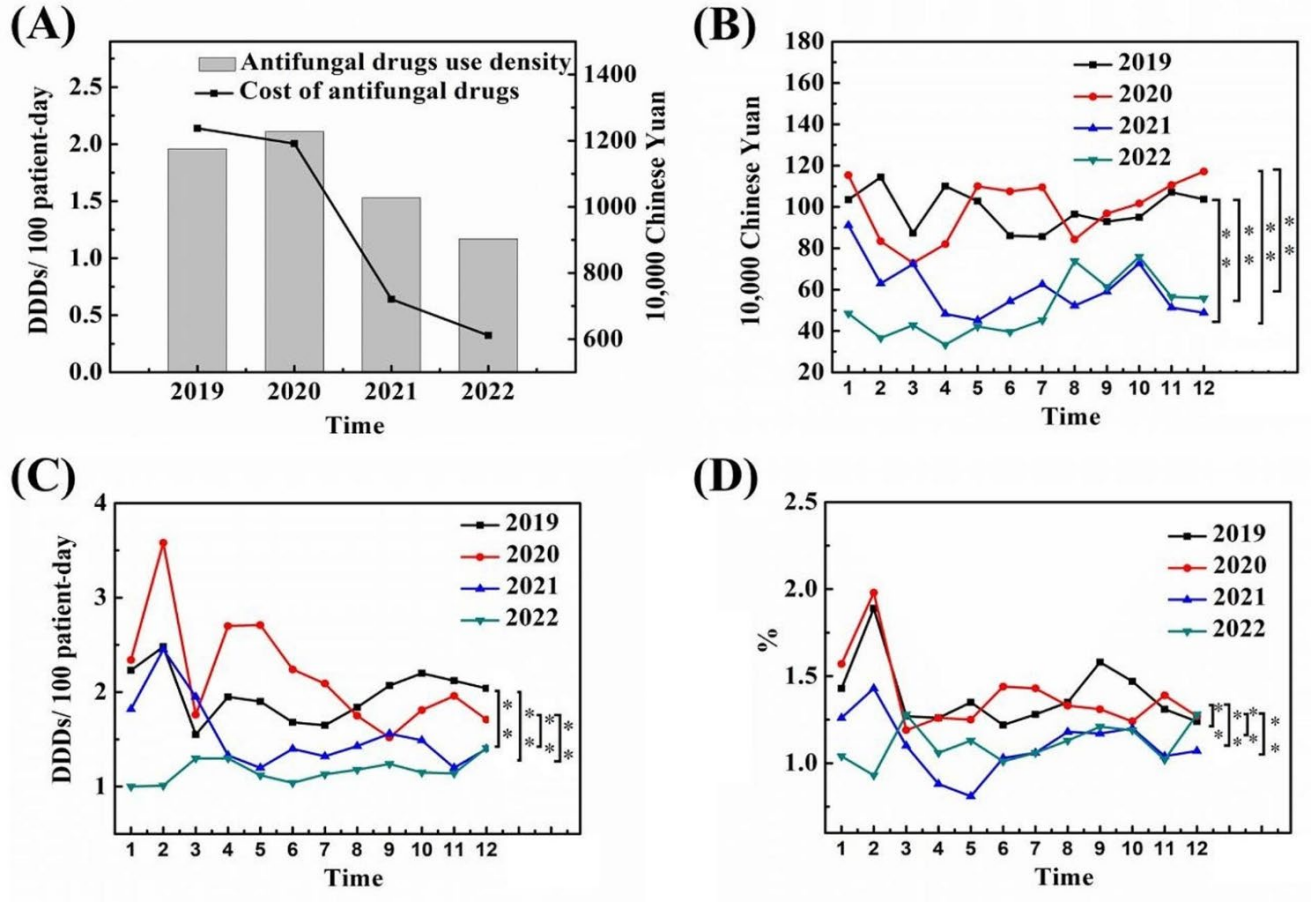
(**A**) The consumption of antifungal agents before and after the AFS intervention expressed in cost and the antifungal drug use intensity. The consumption of antifungal agents per month expressed in (**B**) cost, (**C**) antifungal drug use intensity, and (**D**) the utilization rate of antifungal drugs. (0.01 ≤ *p < 0.05, **p < 0.01)

The consumption of antifungal drugs before and after the implementation of AFS in the pilot hospital was recorded as the cost of antifungal drugs, antifungal drug use intensity, and utilization rate of antifungal drugs, which was recorded once a month. Figure 3 shows that there was no significant difference in the consumption of antifungal drugs between 2019 and 2020, which was significantly reduced after the implementation of AFS. Compared to the date before intervention (2019), the antifungal drug use intensity after intervention (2022) decreased from 1.96 to 1.17 DDDs/100 patient-days, a decrease of up to 40.93%. Moreover, the cost of antifungal drugs decreased from 11.85 million yuan to 6.11 million yuan.

**Fig. 4:**
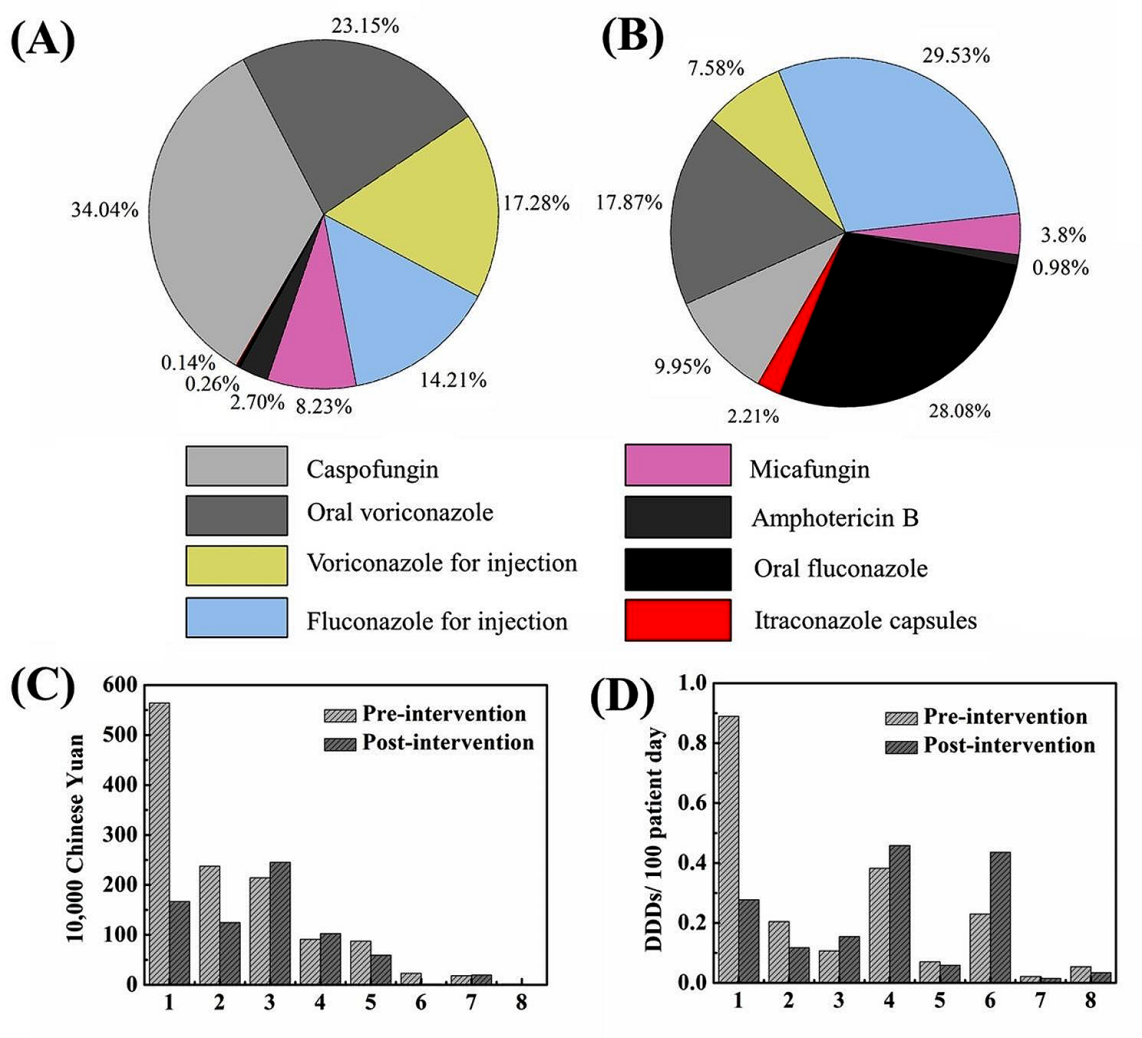
The proportion of the consumption of different antifungal agents after AFS intervention calculated in (**A**) cost and (**B**) antifungal drug use intensity. The consumption of different antifungal drugs pre- and post-intervention calculated in cost (**C**) and (**D**) the antifungal drug use intensity: (**1**) oral voriconazole, (**2**) voriconazole for injection, (**3**) caspofungin, (**4**) fluconazole for injection, (**5**) micafungin, (**6**) oral fluconazole, (**7**) amphotericin B, (**8**) itraconazole capsules

Subsequently, the changes in the consumption of various antifungal drugs were analysed after the implementation of the AFS programme. As shown in Fig. 4, voriconazole accounted for the highest proportion of the total consumption of antifungal drugs calculated by cost in 2021 (40%), followed by caspofungin (34%). Based on the intensity of antifungal drug use, relatively inexpensive fluconazole accounted for the largest proportion of antifungal drugs, exceeding 50%, followed by voriconazole. After AFS intervention, the consumption of voriconazole decreased compared to before, while the consumption of fluconazole and carpofungin showed an upward trend.

### Prescription quality assessment

**Fig. 5:**
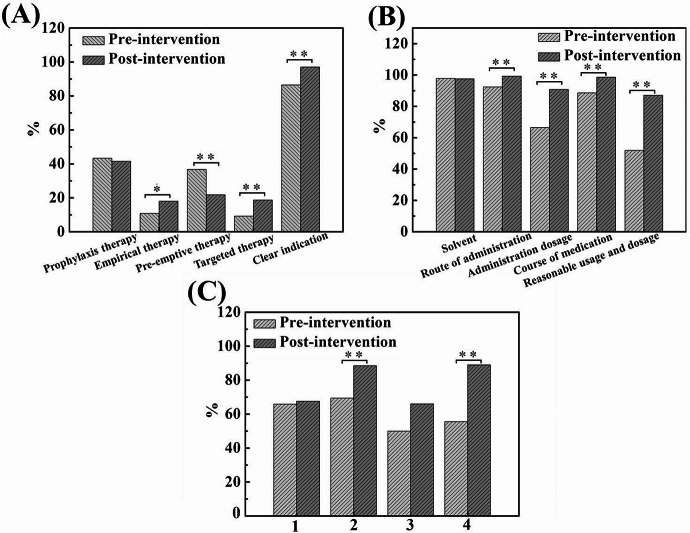
(**A**) Proportion of antifungal drug prescriptions with different indications pre- and post-intervention. (**B**) Proportion of rational prescriptions by usage and dosage. (**C**) (**1**) The combined detection rate of the G test and GM test and (**2**) the combined testing rate of fungal culture and smear microscopy before the use of voriconazole. (**3**) The detection rate of G test and (**4**) the detection rate of fungal culture before the use of echinocandins. (0.01 ≤ *p < 0.05, **p < 0.01)

Clinical pharmacists specializing in anti-infective agents were responsible for reviewing the rationality for prescribing antifungal drugs. A total of 214 cases treated with antifungal drugs were reviewed during the baseline survey period, and 303 cases were reviewed after AFS intervention. As shown in Figs. 5A and 86.4% of the antifungal drug prescriptions met indications in 2019, with the main problem being nonindicated fluconazole and voriconazole prescriptions. Among all prescriptions with indications, prophylaxis therapy was the most common indication (43.2%), followed by preemptive therapy (36.8%). However, the proportion of targeted therapy for fungal infections was less than 10%. After the implementation of AFS, the proportion of antifungal drugs prescribed with indications increased significantly (97.0%). Although the most common indications for these the prescriptions were still prophylaxis therapy and preemptive therapy, the proportion obviously decreased. In particular, the proportion of targeted therapy significantly increased to 18.7%, which was more than twice the amount before AFS. The deficiencies in the usage and dosage of antifungal drugs mainly included inappropriate solvent choices, inappropriate administration routes, nonstandard dosages and unreasonable durations of medication, which were listed in Fig. 5B. Approximately 50% of prescriptions were unreasonable in terms of usage and dosage during the baseline survey period, with approximately 40% of prescriptions showing nonstandard dosages. The most common problem was the nonstandard load dose of voriconazole. After the implement of AFS, the proportion of reasonable prescriptions for antifungal drugs increased to 87.1%, 1.68 times greater than before.

In addition, the microbial detection rate before the use of antifungal drugs in the reviewed prescriptions was calculated. For voriconazole, the combined detection rates of the G test and GM test, as well as the combined detection rates of fungal culture and smear microscopy, were calculated. For echinocandins, the detection rates of the G test and fungal culture before administration were calculated. Figure 5C shows that the rate of fungal cultures before the use of antifungal drugs is significantly greater after the AFS intervention than before (*p* < 0.01).

### Microbiological examinations and results

**Fig. 6:**
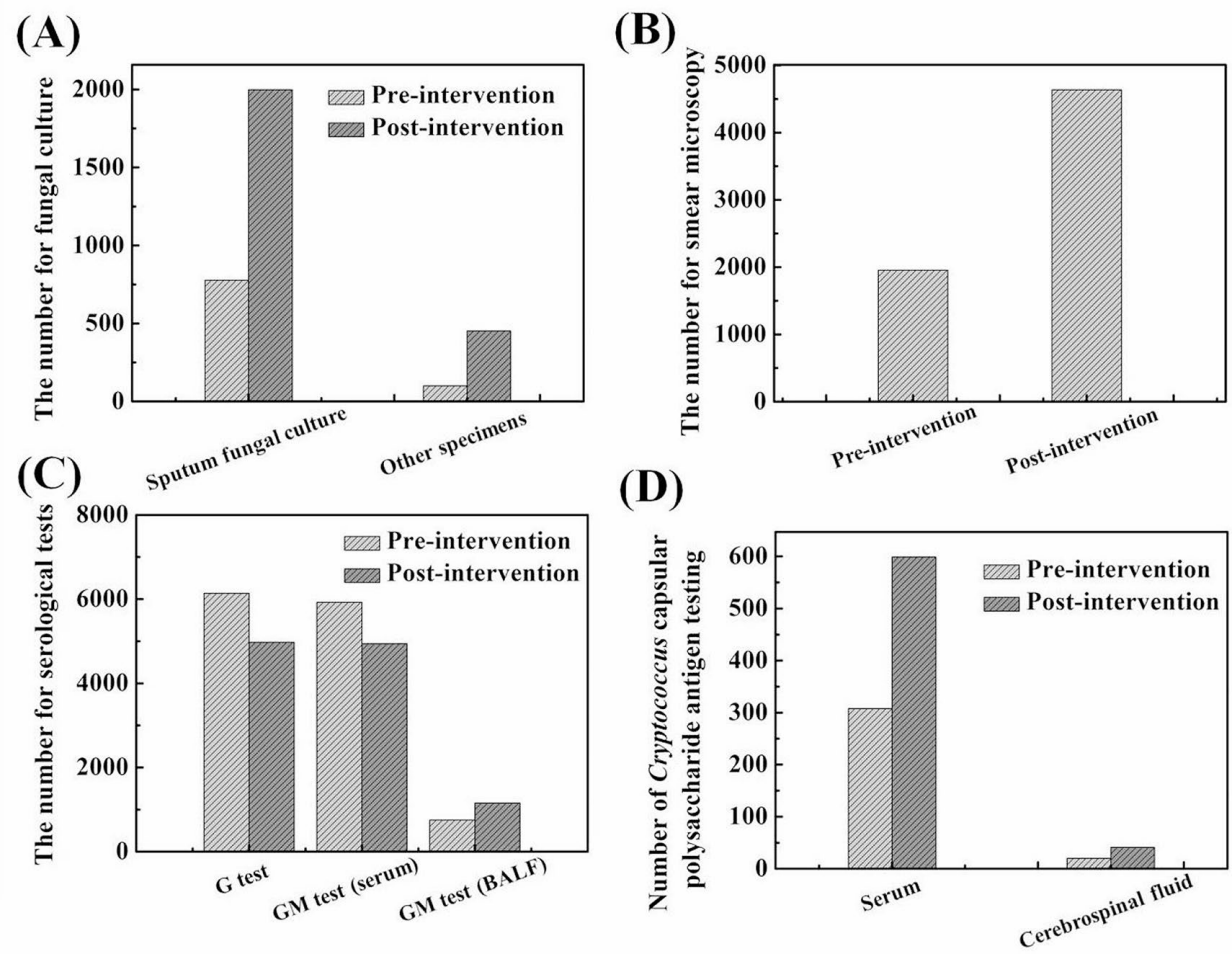
The number of samples submitted for (**A**) fungal culture, (**B**) smear microscopy, (**C**) *Aspergillus* related serological testing, and (**D**) *Cryptococcus* capsular polysaccharide antigen testing pre and post AFS intervention

In addition to the microbiological testing rate before the use of antifungal drugs in the reviewed prescriptions, the number of microbial examinations performed in the entire hospital was summarized to reflect the situation of microbial examination from a macro perspective. As shown in Fig. 6, fungal cultures were dominated by sputum specimen cultures. In 2019, 777 sputum samples were obtained for fungal culture, while 100 other specimens (such as blood, urine, and ascites) were obtained. In 2022, the number of specimens submitted for fungal culture increased significantly compared to that before, with the number of sputum specimens increased to 1999, which was 2.5 times that before AFS intervention, and the number of fungal cultures of other specimens reached 452, which was 4.5 times that of before AFS intervention. In addition to fungal culture, microscopic examination also showed a gradual upward trend over time. There was no significant increase in the number of samples submitted for the G test or serum samples for the GM test before and after the implementation of AFS. However, the number of BALF samples submitted for GM testing and the number of samples submitted for the detection of *Cryptococcus* capsular polysaccharide antigen showed an upward trend.

The positive results were collected from the microbiological laboratory to analyse the situation of fungal infections in pilot hospital and the progress in fungal resistance. As shown Fig. S3, the positive number of fungal cultures, smear microscopy and serological tests after the implementation of AFS was greater than that before AFS. Among all the isolated fungi, *Candida* and *Aspergillus* accounted for the majority. In addition, fungi such as *Mucorales*, *Cryptococcus neoformans*, *Talaromyces marneffei* and other fungi were also isolated. In addition, the composition of *Aspergillus* and *Candida* was analysed. *Aspergillus fumigatus*, *Aspergillus flavus*, *Aspergillus niger* and *Aspergillus terreus* were the main *Aspergillus* detected both before and after the implement of AFS, and *Aspergillus fumigatus* was the predominant fungus. *Candida albicans*, *Candida glabrata*, *Candida krusei*, *Candida parapsilosis* and *Candida tropicalis* were the main *Candida* detected both before and after the implement of AFS, and *Candida albicans* was the predominant fungus. There was a decrease in the proportion of fluconazole-resistant *Candida* isolates from 13.73 to 10.12% after the intervention, and the difference was not statistically significant.

## Discussion

In recent years, the incidence of IFDs has increased significantly, and IFDs are associated with high patient mortality; moreover, the high cost of antifungal drugs has created a substantial economic burden. To promote the standardized use of antifungal drugs and solve the problems of insufficient diagnostic capabilities for fungal infections, poor patient prognosis, and increased fungal resistance, this study explored the scientific practice method of AFS project combined with the PDCA cycle in a tertiary first-class hospital in China and evaluated its effectiveness. After antibacterial drug management led by the National Health Commission of China for more than 10 years, the pilot hospital achieved outstanding results and established a mature AMS system, laying a good foundation for the development of AFS [27].

Prior to the implementation of AFS, a baseline survey was conducted at the pilot hospital to identify the main challenges and issues associated with the use of antifungal drugs and to determine the entry points for the development of AFS. According to the baseline data, the overall consumption of antifungal drugs in pilot hospitals was high. While the positive number of relevant microbiological examinations in some key departments with the greatest antifungal drug use (the department of haematology and the department of organ transplantation) was not consistent with the high consumption of antifungal drugs. There may have been a problem of “neglecting diagnosis and valuing treatment” in antifungal treatment, causing the unreasonable use of antifungal drugs. The main reasons were as follows. (1) The management system for the rational application of antifungal drugs was not sound, and the division of responsibilities in various departments was not clear. (2) The degree to which clinicians emphasized the standard use of antifungal drugs was low, and the professional knowledge of these doctors was insufficient. (3) The information system was defective and unable to realize the prereview mechanism for the use of antifungal drugs. (4) The relevant management system, assessment intensity as well as reward and punishment policies were not clear. Taking these problems as the focus, targeted control plans with measures were developed, and AFS was carried out.

The implementation of AFS has standardized the clinical use of antifungal drugs in the pilot hospital, as it has shown remarkable effects in reducing the amount, use intensity and utilization rate of antifungal drugs. AFS was expected to save 5.74 million yuan in the cost of antifungal drugs per year, which was significantly higher than the potential savings observed in other studies [28, 29]. This indicated that there was a large room for improvement in the use of antifungal drugs in pilot hospital before the intervention, and validated that the implementation of systematic and strict AFS was economical, which could considerably prevent the abuse of antifungal drugs and reduce medical costs. Compared with other studies where there was no significant change in the use intensity of antifungal drugs [13], this study significantly reduced the use intensity of antifungal drugs after AFS intervention, indicating that the reduction in the cost of antifungal drugs was not only achieved through the use of low-priced drugs, but actually reduced drug consumption. Specifically, we compared the consumption of various antifungal drugs before and after the implementation of AFS. The reduction of the consumption for voriconazole and the increase of the consumption for fluconazole and carpofungin were more suitable for the situation in which the detection rate of *Candida* was higher than that of *Aspergillus* in the pilot hospital [20], fully reflecting that the development of AFS could not only reduce the consumption of antifungal drugs overall but also play a corrective role in the selection of antifungal drugs in the clinic.

The inappropriate rates of antifungal drug prescriptions decreased significantly from 55.14 to 15.51% after the intervention of AFS. This trend was comparable to some literature reports [30, 31]. Before the implementation of AFS, the main reasons for inappropriate prescription of antifungal drugs included nonindicated prophylactic drug use and insufficient refinement of antifungal drug usage, reflecting the lack of clinical awareness and diagnostic ability regarding invasive fungal diseases. Some of these issues could be avoided by modifying the content of the pre-examination system based on information technology to promote the standardized use of antifungal drugs. After the implementation of AFS, the proportion of antifungal prescriptions with indications increased significantly, and the proportion of prescriptions with appropriate administration routes, reasonable dosage, and standardized treatment courses significantly increased. The unnecessary consumption of antifungal drugs could be reduced by avoiding the use of antifungal drugs without indications and increasing the awareness of clinical physicians in terms of accurate diagnosis. Optimizing the usage and dosage of antifungal drugs was expected to improve the treatment efficacy for fungal infections, curb the progression of fungal resistance and the occurrence of adverse drug reactions, and improve the therapeutic efficacy for patients, which was of great significance for ensuring medical quality and medical safety [32]. Although the proportion of targeted therapy of antifungal drugs has significantly increased after AFS intervention, there is still some room for improvement compared with other studies [33]. The decisions regarding the use of antifungal drugs for prophylaxis therapy and empirical therapy need to be further optimized in clinical practice, taking into account the specific situation of patients [34].

The results of microbiological tests, including serological tests and fungal cultures, are necessary indicators for the probable diagnosis and proven diagnosis of IFDs. The guide to the utilization of the microbiology laboratory for the diagnosis of infectious diseases updated in 2018 by the IDSA emphasized the necessity of microbiological testing and clearly stated the requirement that specimens should be collected prior to the use of antibacterial agents to clarify the diagnosis and adjust the medication regimen according to the results of drug susceptibility tests [35]. After the implementation of AFS, the microbial detection rate before the use of antifungal drugs increased significantly, suggesting that the awareness of clinicians for retaining suitable specimens to improve etiological detection significantly increased. The decrease in the proportion of fluconazole-resistant *Candida* isolates after the intervention of AFS reflected the importance of AFS in prolonging the service life of antifungal drugs.

On the basis of AFS, this study introduced the use of the PDCA quality management tool to manage the use of antifungal drugs. Through the continuous improvement of the management system and working mechanism, not only was the consumption of medical resource in our hospital significantly reduced, but the diagnostic and therapeutic capabilities of medical staff on fungal infections were also enhanced. The irrational use of antifungal drugs was greatly reduced. By taking the lead in carrying out AFS, the pilot hospital has established an effective management model, set up a professional management team and a multidisciplinary professional technical team, and created a set of diagnostic and treatment standards suitable for local area, marking the transition of antifungal drug management to be scientific, standardized, refined and normalized. However, this study has several limitations. First, this study was conducted in a tertiary first-class hospital, and the results were not sufficient to represent an overview of other medical institutions. In addition, it should be noted that the pilot hospital did not carry out therapeutic drug monitoring for antifungal drugs during the study period, and the samples needed to be tested for the blood concentration of antifungal drugs were sent to other hospitals for testing. It restricted the development of personalized treatment plans for patients and the evaluation of the rationality of the dosage to some extent. In summary, AFS was recommended for implementation in pilot hospitals, and its effectiveness in regulating the use of antifungal drugs was verified. Therefore, it is necessary to further enrich the practice of AFS, explore antifungal drug management systems in line with the national conditions of various countries, and study the clinical value of AFS on a larger scale.

## Conclusions

This study reported on the innovative proposal and exploration of AFS using the PDCA quality management tool in a tertiary first-class hospital in China. It demonstrated that it was feasible to reduce the consumption of antifungal drugs, increase the rational use of antifungal drugs, and improve the awareness of microbiological examination among clinicians through the systemic implementation of AFS. This practice is an effective means of continuously improving the rational use of antifungal drugs and reducing the development of fungal resistance, providing a replicable reference strategy for other health care systems.

### Electronic supplementary material

Below is the link to the electronic supplementary material.

Supplementary Material 1

## Data Availability

The datasets for the study are available from the corresponding author upon request.

## Abbreviations

AFS: Antifungal stewardship
PDCA: Plan-do-check-act
AMS: Antimicrobial stewardship
IFDs: Invasive fungal diseases
CLSI: Clinical and Laboratory Standards Institute
HIS: Hospital information system
IDSA: Infectious Diseases Society of America
ESCMID: European Society of Clinical Microbiology and Infectious Diseases
G test: 1,3-β-D-glucan test
GM test: Galactomannan test
DDDs: Defined daily doses
BALF: Bronchoalveolar lavage fluid
