# Supplementary material for: The practice and evaluation of antifungal stewardship programs at a tertiary first-class hospital in China
Source: BMC Infect Dis. 2024 May 21;24:506. doi: 10.1186/s12879-024-09405-x (PMC11106957; doi:10.1186/s12879-024-09405-x)
Supplement: Supplementary file 1 — Supplementary Material 1 [file 12879_2024_9405_MOESM1_ESM.docx]

**The practice and evaluation of antifungal stewardship programs at a tertiary first-class hospital in China**

Huiyuan Zhang^1†^, Yinglin Wang^1†^, Ruigang Diao^1^, Xuechen Huo^2^*, Quan Zhao^1^*

**Methods**

**Hierarchical management system for antifungal drug prescriptions**

The pilot hospital has developed a hierarchical management system for antifungal drugs. Antifungal drugs were classified into three levels based on the safety, efficacy, fungal resistance, price and other factors: unrestricted use grade (such as oral fluconazole), restricted use grade (such as oral voriconazole, fluconazole for injection) and special use grade (such as voriconazole for injection, amphotericin B, caspofungin). Physicians with intermediate or higher professional and technical qualifications could be granted the right to prescribe restricted grade antifungal drugs after receiving unified antifungal drug training and passing assessments, while physicians with senior professional and technical qualifications couldbe granted the right to prescribe special use grade antifungal drugs after that they have been trained and passed the examination. What’s more, the clinical application of special grade antifungal drugs required strict adherence to medication indications, which could be prescribed by physicians with corresponding prescription rights only after the consultation and approval by a professional clinical pharmacist or infectious disease specialist.

**Education and training content**

The training content during the implement of AFS included but was not limited to the risk identification of fungal infection, the diagnosis and treatment of fungal infectious diseases, the rational use of antifungal drugs, methods for correct retention of specimens and microbial detection methods, the correct interpretation of drug sensitivity results, the popular pathogenic spectrum of fungi in each department, the analysis of common errors in antifungal drug use at various clinical departments, diagnosis and treatment strategies for special patients (such as organ transplant recipients and patients with hematological disorders), and the medical insurance reimbursement for antifungal drugs.

**Clinical application management indices of antifungal drugs**

Clinical application management indices of antifungal drugs were designed systematically and included the following aspects: (1) statistics and ranking of the utilization rate of antifungal drugs among inpatients in the whole hospital and key departments; (2) statistics and ranking of the use intensity of antifungal drugs among inpatients in the whole hospital and key departments; (3) statistics and ranking of the consumption amount of antifungal drugs in inpatients in the whole hospital and key departments; (4) the proportion of the amount of essential antifungal drugs used in the whole hospital and key departments; (5) the rational rate of antifungal drug prescriptions in each department; (6) the combined submission rate and positive rate of G test and GM test before the use of voriconazole; (7) the combined submission rate and detection rate of fungal culture and fungal smear test before the use of voriconazole; (8) the submission rate and positive rate the G test before the use of echinocandins; (9) the submission rate and detection rate of fungal culture and smear tests before the use of echinocandins; (10) ranking of departments based on the use intensity and amount of use for each category of antifungal drugs; and (11) the incidence of invasive candidiasis and disseminated candidiasis in the whole hospital and key departments. The above management indicators required statistical evaluation every three months.

**The standardized checklist for antifungal therapy**

As shown in Table S1, the management team of AFS designed and developed a periodic evaluation table of AFS management effectiveness for clinical departments to score the rationality of antifungal drugs, which was performed every three months.

**Table S1** The periodic evaluation table of AFS management effectiveness for clinical departments

| Department | | Score |
| --- | --- | --- |
| Number of patients prescribed antifungal drugs | |  |
| Compliance rate with guidelines | |  |
| Drug selection (indication) | Number of patients receive prophylaxis therapy |  |
|  | Number of patients receive empirical therapy |  |
|  | Number of patients receive preemptive therapy |  |
|  | Number of patients receive targeted therapy |  |
| Rational rate in usage and dosage | Rational rate in solvent selection |  |
|  | Rational rate in administration route |  |
|  | Rational rate in administration dosage |  |
|  | Rational rate in drug duration |  |
| Number of patients undergoing de-escalation therapy | |  |
| Number of patients undergoing sequential oral therapy | |  |
| Microbiological testing | Combined detection of the G test and GM test before the use of voriconazole |  |
|  | Combined detection of fungal culture and smear microscopy before the use of voriconazole |  |
|  | Detection of G test before the use of echinocandins |  |
|  | Detection of fungal culture before the use of echinocandins |  |
| Incidence of candidaemia | |  |
| Continuous time of positive culture/time before turning negative | |  |
| Number of recurrent fungal infections | |  |
| Number of breakthrough infections | |  |
| Mortality from fungal infections | |  |

**Results**


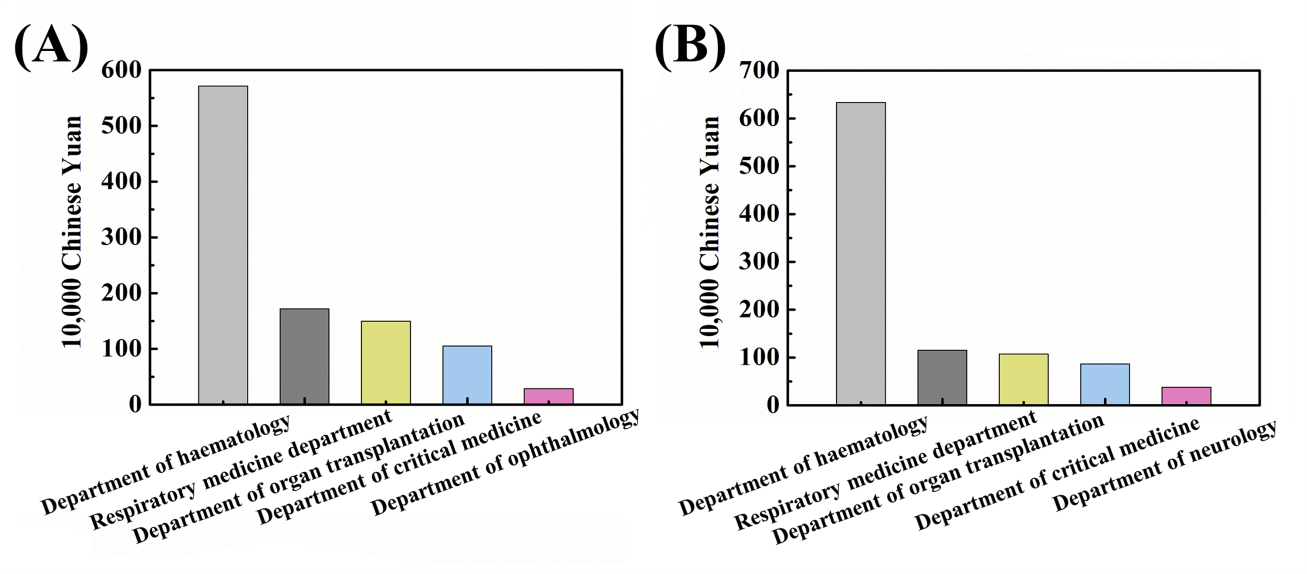


**Fig. S1** The top five departments with the greatest antifungal drug use in 2019 (A) and in 2020 (B).

**Table S2** The number of positive microbiological results in key departments at the first half year of 2020.

| Department | *Candida* culture | *Aspergillus* culture | Smear microscopy | GM test (serum) | GM test (BALF) |
| --- | --- | --- | --- | --- | --- |
| Haematology | 28 | 3 | 1 | 22 | 0 |
| Respiratory medicine | 49 | 26 | 15 | 4 | 91 |
| Organ transplantation | 7 | 0 | 0 | 3 | 0 |
| Critical medicine | 39 | 0 | 0 | 23 | 7 |

**
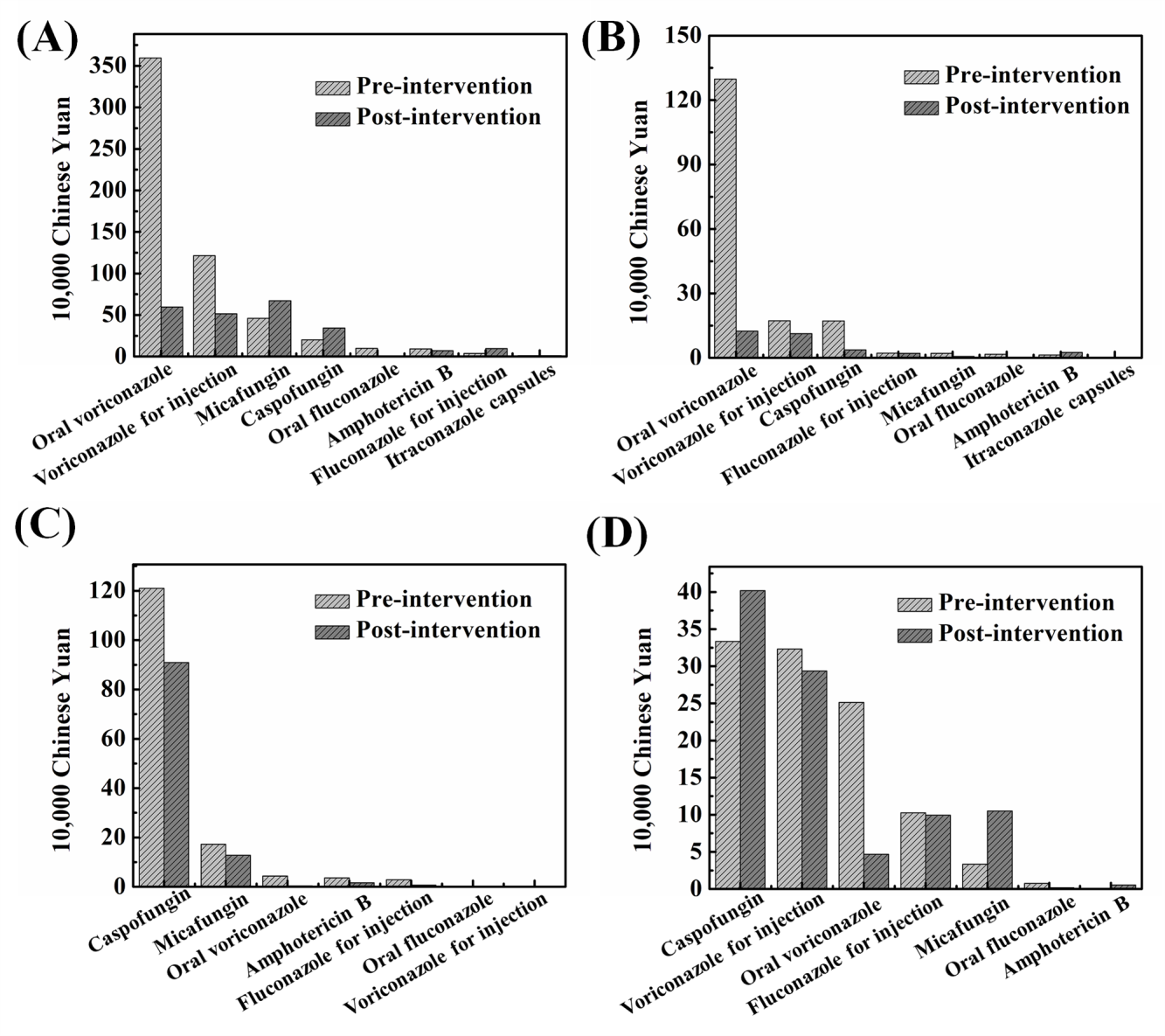
Fig. S2** The consumption of different antifungal drugs pre- and post-intervention calculated in cost in (A) the department of haematology, (B) the respiratory medicine department, (C) the department of organ transplantation and (D) the department of critical medicine.


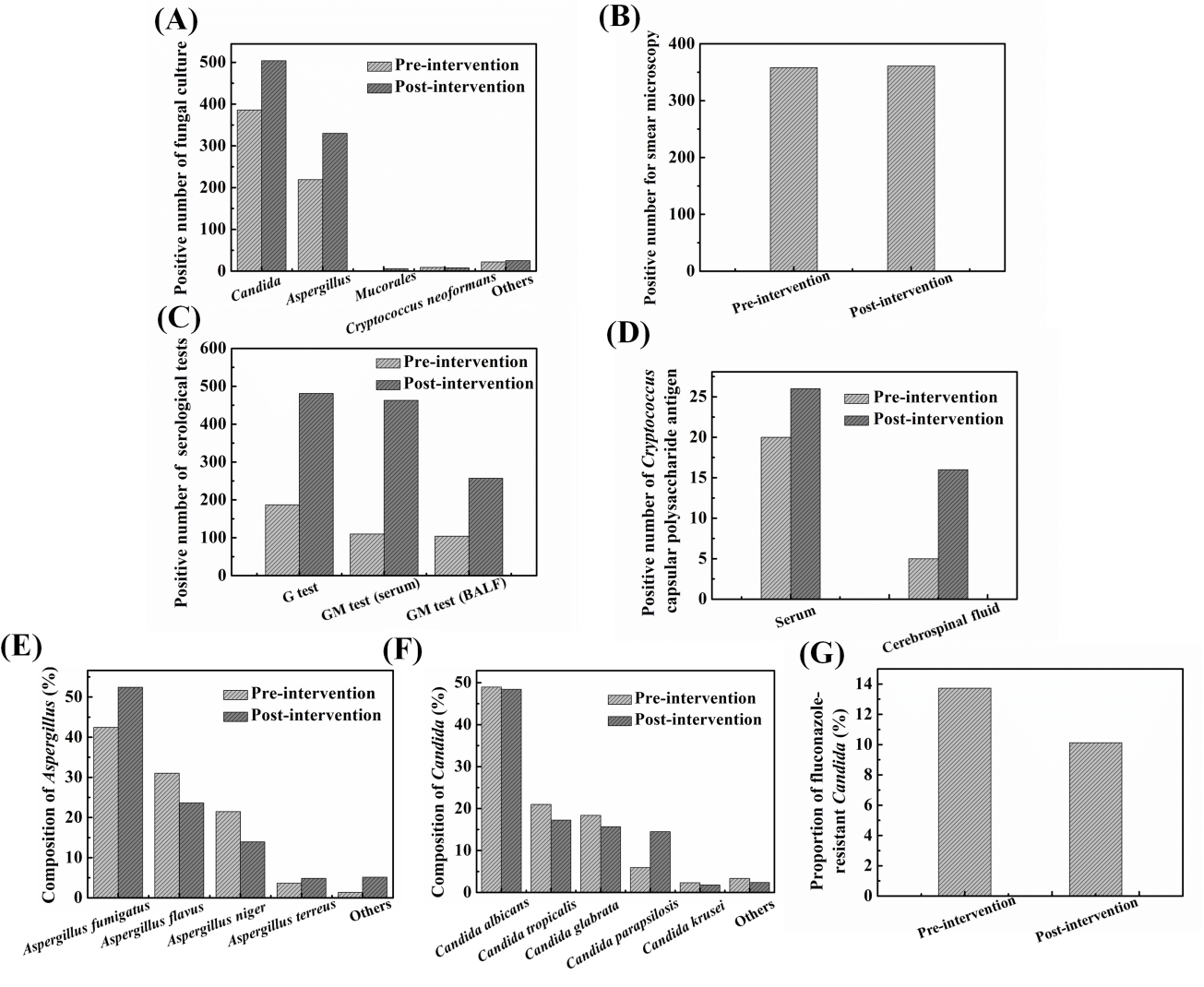


**Fig. S3** The positive number of (A) different fungi identified, (B) smear microscopy, (C) *Aspergillus*-related serological tests, (D) *Cryptococcus* capsular polysaccharide antigen examination before and after the implement of AFS. (E) The composition of *Aspergillus* identified, (F) the composition of *Candida* identified and (G) the proportion of fluconazole-resistant *Candida* isolates.
